# The solvation of electrons by an atmospheric-pressure plasma

**DOI:** 10.1038/ncomms8248

**Published:** 2015-06-19

**Authors:** Paul Rumbach, David M. Bartels, R. Mohan Sankaran, David B. Go

**Affiliations:** 1 Department of Aerospace and Mechanical Engineering, University of Notre Dame, Notre Dame, Indiana 46556, USA; 2 Department of Chemistry and Biochemistry, Notre Dame Radiation Laboratory, University of Notre Dame, Notre Dame, Indiana, 46556, USA; 3 Department of Chemical and Biomolecular Engineering, Case Western Reserve University, Cleveland, Ohio, 44106, USA; 4 Department of Chemical and Biomolecular Engineering, University of Notre Dame, Notre Dame, Indiana 46556, USA

## Abstract

Solvated electrons are typically generated by radiolysis or photoionization of solutes. While plasmas containing free electrons have been brought into contact with liquids in studies dating back centuries, there has been little evidence that electrons are solvated by this approach. Here we report direct measurements of solvated electrons generated by an atmospheric-pressure plasma in contact with the surface of an aqueous solution. The electrons are measured by their optical absorbance using a total internal reflection geometry. The measured absorption spectrum is unexpectedly blue shifted, which is potentially due to the intense electric field in the interfacial Debye layer. We estimate an average penetration depth of 2.5±1.0 nm, indicating that the electrons fully solvate before reacting through second-order recombination. Reactions with various electron scavengers including H^+^, NO_2_^−^, NO_3_^−^ and H_2_O_2_ show that the kinetics are similar, but not identical, to those for solvated electrons formed in bulk water by radiolysis.

## Abstract (web summary)

Free, or solvated, electrons in a solution are known to form at the interface between a liquid and a gas. Here, the authors use absorption spectroscopy in a total internal reflection geometry to probe solvated electrons generated at a plasma in contact with the surface of an aqueous solution

## Graphical abstract

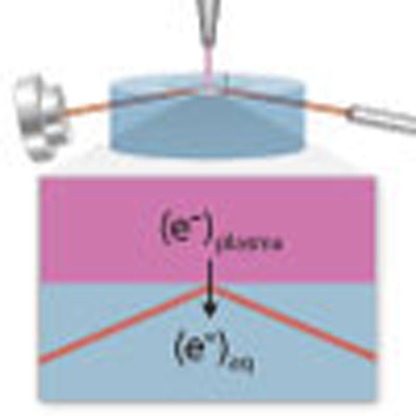

Because of their superlative reducing power and fascinating unresolved questions regarding their structure, solvated electrons remain a very active subject of research. Dating back to the initial discovery of the solvated electron in 1962 (1,2), electron radiolysis, which uses a high-energy (relativistic) beam of electrons to ionize water in the bulk^3^, has long been a workhorse for studying solvated electrons. Along with laser photolysis^4^, it has been instrumental in advancing our understanding of the physical mechanisms behind ionic solvation and free radical chemistry in aqueous solutions. Recently, new approaches to generate and study solvated electrons have emerged, including low-energy photoemission from submerged diamond^5^ and via an effusive sodium beam in vacuum^6^. Of particular interest is the near-surface region between liquid and vacuum or liquid and gas, and a number of studies have suggested that electrons may only partially solvate at these interfaces in certain cases^7^,^8^,^9^,^10^, with some of the electron density projecting out of the liquid into the vacuum or gas. However, experimentally probing near-surface solvated electrons is inherently challenging, and their properties remain poorly understood.

Gas discharges (plasmas) provide an interesting alternative for generating near-surface solvated electrons in water. For well over a century, plasmas have been brought into contact with liquids to explore the possibility of bringing charged species, including electrons, from the gas into the liquid phase^11^. Yet, there has been only indirect evidence that electrons traverse the plasma–solution interface and become solvated, where scavenging reactions have been used to measure long-lived products in the bulk^12^.

Here we present a highly sensitive optical technique that allows us to directly probe the plasma–solution interface for short-lived chemical species, and report the first direct spectroscopic evidence of solvated electrons generated by an atmospheric-pressure plasma near the surface of aqueous solutions. We detect solvated electrons by their optical absorption and characterize their average penetration depth and reactivity, where high sensitivity at the plasma–solution interface is achieved by a total internal reflection geometry and lock-in amplification of a modulated plasma current. Our measurements show that plasma electrons fully solvate in the bulk before reacting away at rates similar to solvated electrons formed by pulse radiolysis. However, we measure a blue-shifted absorption spectrum that indicates that the solvation at a plasma–solution interface is distinct from other environments, which can potentially be attributed to the high interfacial electric field that forms at the interface.

## Results

### Measurements of the optical absorption spectrum

**Figure 1:**
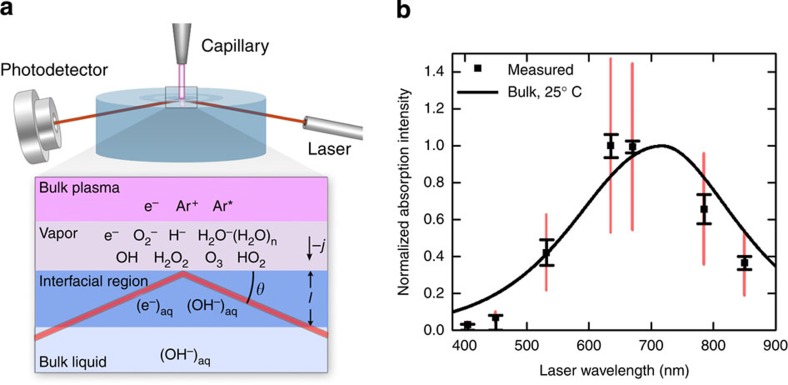
Generation and detection of solvated electrons by an atmospheric-pressure plasma. (**a**) Schematic of experimental apparatus for optical detection using a total internal reflection configuration. Anticipated chemical species in the different phases are also shown. Note: the submerged counter-electrode has been omitted from this figure to emphasize the optical measurement. (**b**) Measured optical absorption signal corresponding to solvated electrons measured at the plasma–solution interface using laser diodes at different wavelengths. Black error bars with capped ends represent the r.m.s. variance in the raw data, and the overlaid red error bars also account for the systematic uncertainty in the laser–plasma overlap. Both sets of error bars represent 90% confidence. A Gaussian-Lorentzian bulk spectrum (solid line) measured in pulse radiolysis experiments for a temperature of 25 °C (ref. 15) is included as a guide.

An atmospheric-pressure plasma in argon was formed at the surface of an aqueous solution by biasing a metal capillary electrode suspended over the solution with negative high voltage relative to a submerged anode (Supplementary Fig. 1). This led to breakdown of the gas between the capillary tip and the surface of the solution, and allowed electrons to flow from the plasma phase into the solution phase. As shown in Fig. 1a, solvated electrons were detected by measuring their strong red absorbance (*λ*_max_∼700 nm) using a total internal reflection configuration, which allowed us to detect their solvation directly at the plasma–solution interface (Supplementary Note 1). According to Beer's law, the normalized optical absorbance is


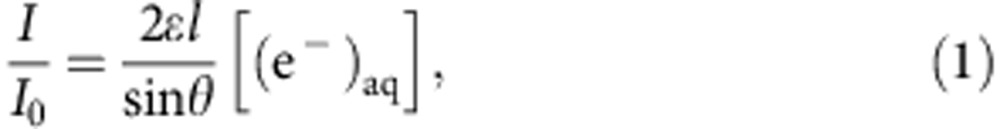


where *I*_0_ is the incident intensity, *θ* is the angle of incidence relative to the liquid surface, *ɛ* is the extinction coefficient, *l* is the average penetration depth and [(e^−^)_aq_] is the average interfacial concentration of solvated electrons. Given the expected short penetration depth of low-energy electrons from the plasma (∼nm)^13^ and their short lifetime (limited to ∼μs by second-order recombination at high concentration)^2^,^14^, we anticipated the production of a very thin film of solvated electrons near the plasma–solution interface, and absorbance on the order of 10^−5^ OD. To measure such a small absorption, a number of steps were taken to increase the signal to noise, most important of which was modulation of the plasma current at 20 kHz to produce a similar modulating absorbance intensity that could be isolated with lock-in detection (Supplementary Figs 2 and 3).

An absorption spectrum at the plasma–solution interface was obtained with a series of individual diode lasers and 163 mM solutions of sodium perchlorate (NaClO_4_), as shown in Fig. 1b. The broad absorption peak in the red generally agrees with pulse radiolysis experiments^15^. However, interestingly, the measured spectrum with a peak near ∼650 nm is blue shifted by ∼50 nm from the spectrum typically observed for bulk solvated electrons at room temperature. This is surprising given that in pulse radiolysis, increased water temperature results in a red shift^15^. We would expect Joule heating at the interface would increase the local temperature at the interface leading to a similar red shift, which we do not observe. Furthermore, the tail in the blue at 405 and 450 nm appears to be suppressed compared with the well-known Lorentzian profile observed in pulse radiolysis experiments. A very small blue shift of ∼5 nm can be attributed to the relatively high salt content of our solutions^16^ but this does not account for the magnitude of the shift observed here. In addition, we cannot explain the very low absorptions measured at 405 and 450 nm in terms of any kind of shift of the bulk spectrum.

There are significant physical differences between bulk water and a plasma–solution interface that could potentially alter the spectrum. In particular, the Debye layer that forms at the plasma–solution interface contains a net space charge and an intense electric field that could result in a second-order Stark shift, affecting both the peak location and the shape of the observed spectrum. For example, the recently discovered quantum-confined Stark effect in semiconductor nanocrystals has been shown to produce blue shifts >10 nm for electric fields ∼10^7^ V m^−1^ (ref. 17)—a field strength commonly found in aqueous Debye layers^18^. Overall, there has been little to no research involving the Stark effect on the solvated electron absorption spectrum, and this should be an area of future study. In addition, recent work has shown that partial solvation may also alter the measured spectrum^10^, although results presented in the following section indicate the electrons solvate well into the bulk.

The error bars shown in black in Fig. 1b reflect the root mean square (r.m.s.) variance of the absorption signal at 90% confidence and highlight the blue shift in the spectrum. However, we point out that variations in the laser spot size for the different diode lasers also contribute to error in the measured spectrum. We calculate a correction factor to the normalized absorption intensity to account for the Gaussian overlap of the plasma (Supplementary Fig. 4; Supplementary Table 1) and the laser spot size, and we conducted a detailed uncertainty analysis of this systematic effect (Supplementary Notes 2,3,4). Although the correction factor only changes the magnitude of each data point by <10%, the associated uncertainties lead to substantial overall combined uncertainty. Thus, in Fig. 1b, we also overlay in red the overall combined uncertainty at 90% confidence, accounting for both the root mean square variation and the systematic effect. We note that the primary contribution to this overall error in the measurements is the uncertainty in the overlap of the plasma with the laser spot (Supplementary Fig. 5), because while the plasma current is precisely controlled and measured, the area of the plasma and the distribution of the current density throughout the area are not known *a priori*. Further, the current density may be an overestimate of the electron flux impinging on the liquid surface, as there could be other charged species contributing to the measured current. Even accounting for these contributions to the overall error in the measurements, the joint probability that the data points at 850 and 785 nm as well as those at 450 and 405 nm all lay on or above the pulse radiolysis spectrum is <1%, indicating that the blue shift is statistically real.

### Extrapolation of the average penetration depth

To estimate the average penetration depth, *l,* from absorption measurements, we consider the reaction kinetics at the interface. In the absence of scavenging species, the solvated electrons quickly react via second-order recombination to form hydroxide and hydrogen via^2^


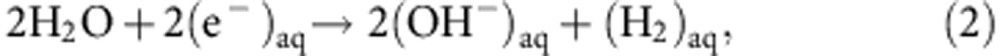


with a rate constant of 2*k*_2_=1.1 × 10^10^ M^−1^ s^−1^ at room temperature^19^. In our previous work, we have measured hydrogen gas from a similar experimental setup using mass spectrometry as well as a pH change associated with the production of hydroxide^20^. Assuming a steady-state balance between the rate at which solvated electrons are created by the plasma, where the volumetric creation rate is proportional to the current density, *j*, and the rate at which they are destroyed via reaction (2), the average concentration of solvated electrons near the surface can be described by (Supplementary Note 2)


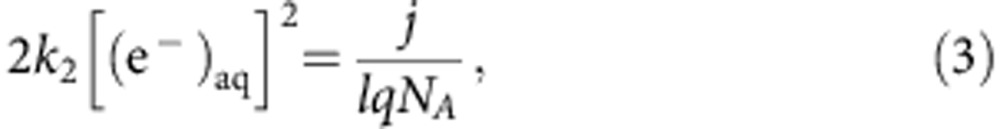


where *q* is the charge of an electron and *N*_A_ is Avogadro's number. By combining equation (3) with equation (1), we can estimate the average penetration depth before the solvated electrons react via (2) from our measured optical absorption signal at 670 nm. Estimating *j* to be the total current divided by the visible area of the plasma for the high and low current states (Supplementary Fig. 4; Supplementary Table 1), and using a value of *ɛ*_670_=1.6 × 10^4^* *M^−1^ cm^−1^ based on radiolysis experiments^21^, we calculated an average penetration depth of *l*=2.5±1.0 nm. The uncertainty of 1.0 nm represents a minimum based on our measurements of the plasma area and the various assumptions in the current density profile (Supplementary Note 5), the same factors that contribute to the large uncertainty in the absorbance measurements. Because these factors are difficult to experimentally measure, we estimate that the uncertainty in *l* could be as large as 2.0 nm. From the current density, we can also estimate the average local concentration of solvated electrons to be ∼1 mM.

In radiolysis experiments, electrons are ejected from water molecules with kinetic energies on the order of tens of eV, and following a random walk trajectory, dissipate their kinetic energy to become solvated by forming a local water shell. The average distance between the site of ionization and eventual electron localization is ∼4 nm (ref. 22). In our experiments, electrons are created in the plasma phase and are accelerated through the anode sheath layer of the plasma, estimated to be ∼10 μm thick based on an electric field of ∼10^6^ V m^−1^ in Ar^23^, towards the water surface. The electric field in this sheath region provides the electrons with an average kinetic energy of ∼1–5 eV (ref. 24) when they impinge on the water surface. Monte Carlo simulations of electrons with incident energies ∼1–10 eV have predicted the average localization depth into liquid water at 25 °C to be 1–10 nm (ref. 13). The average penetration depth of *l*=2.5±1.0 nm we report includes both the distance it takes the ∼1–5 eV electrons to localize as well as the average distance they drift-diffuse before reacting away via reaction (2). Our value for *l* indicates that the near-surface solvated electrons produced by the plasma persist into the bulk of the solution, several monolayers below the surface. It also sets an upper limit for the localization depth of electrons injected by plasma under our conditions.

In general, there are currently two schools of thought on the behaviour of a solvated electron at a solution interface. In the partially solvated view, the electron is assumed to be only at the interface, with some of its electron density projecting into vacuum, and the rest interacting with a few water molecules, which hold the electron at the surface by their dipoles. In the fully solvated view, the electron is fully submerged and resides within a ‘cavity', surrounded by water molecules^25^, although recently the ‘cavity' model has been strongly challenged in favour of a plum pudding model where water is densified by electrostriction, rather than being expelled from the centre^26^,^27^. Irrespective of the model, moment analysis of the experimental absorption spectrum provides a radius of gyration of ∼0.25 nm for the bulk solvated electron^15^. Assuming a water molecule diameter of ∼0.275 nm, this indicates that the depth of the electron must be more than ∼0.525 nm to be considered fully solvated. Our estimation of the penetration depth indicates that these plasma-injected, near-surface solvated electrons are in fact fully solvated. This conclusion is consistent with recent findings of Sagar *et al.* who used laser dissociation of I^−^ to show that electrons are fully solvated at a water–air interface but reside only ∼1 nm from the water surface^28^.

### Measurements of reaction kinetics

We also studied the kinetics of the solvated electrons generated by an atmospheric-pressure plasma by carrying out reactions with various chemical species. Solvated electrons can reduce a wide variety of cations, anions and neutral species and these reactions typically have the form


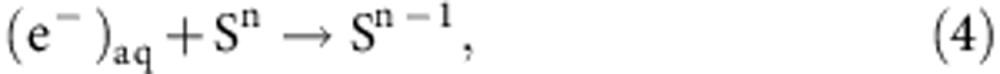


where S is the electron scavenger. Introducing a sufficiently high concentration of scavengers to the solution will lower the equilibrium concentration of solvated electrons, which, in turn, will decrease our optical absorption signal. Using cationic (H^+^), anionic (NO_2_^−^ and NO_3_^−^) and neutral scavengers (H_2_O_2_), we found that the optical absorption signal is rapidly attenuated for sufficiently large scavenger concentrations. For all experiments, a non-reactive background of either NaOH or NaClO_4_ was used to maintain a solution conductivity of at least 2.4 mS cm^−1^, which is necessary for stable operation of the plasma.

**Figure 2:**
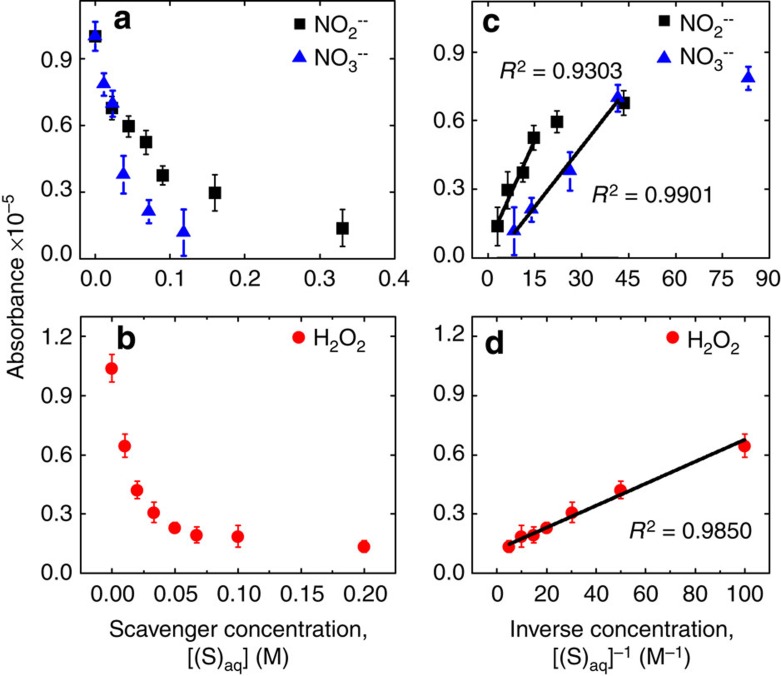
Absorbance behaviour for different scavengers. (**a**) Absorbance measurements for anionic scavengers NO_2_^−^ and NO_3_^−^. (**b**) Absorbance measurements for neutral scavenger H_2_O_2_. (**c**) Corresponding absorbance as a function of the inverse concentration [(S)_aq_]^−1^ for NO_2_^−^ and NO_3_^−^, where the solid lines are linear curve fits. (**d**) Corresponding absorbance as a function of the inverse concentration [(S)_aq_]^−1^ for H_2_O_2_, where the solid line is a linear curve fit. Error bars represent the r.m.s. variance of the absorption signal at 90% confidence.

Assuming a sufficiently high scavenger concentration, the scavenging in reaction (4) will dominate second-order recombination in reaction (2), and the optical absorption signal becomes inversely proportional to the scavenger concentration (Supplementary Note 6). In Fig. 2, results are shown for the anionic scavengers NO_2_^−^ and NO_3_^−^ (Fig. 2a) and the neutral scavenger H_2_O_2_ (Fig. 2b). Apply linear regression analysis to the normalized absorption intensity as a function of inverse scavenger concentration [(S)_aq_]^−1^ (Fig. 2c,d), it is possible to obtain the effective rate constant for each scavenging reaction, which are summarized in Table 1. As with the extrapolated penetration depth, the uncertainty in the measured current density dominates the uncertainty in the extrapolated rate constants. Our values are on the same order of magnitude as values determined from pulse radiolysis experiments^17^, which are also shown in Table 1, but differ by as much as 60%. The literature rate constants for the anion scavengers are corrected using the Brønsted–Bjerrum equation^17^ because our solutions have relatively high ionic strength, *I*_S_, where Debye screening can significantly impact rate constants.

For the case of H_2_O_2_ shown in Fig. 2b,d, the scavenging reaction cited in Table 1 produces the highly reactive OH radical, which can subsequently scavenge additional electrons and recombine to form additional H_2_O_2_. At large concentrations of H_2_O_2_, these reactions will likely result in significant deviations from our simple model. The small values we obtained relative to the literature may be due to depletion of the scavenger concentrations at the interface, both from these reactions and from transport induced by the electric field gradient.

**Figure 3:**
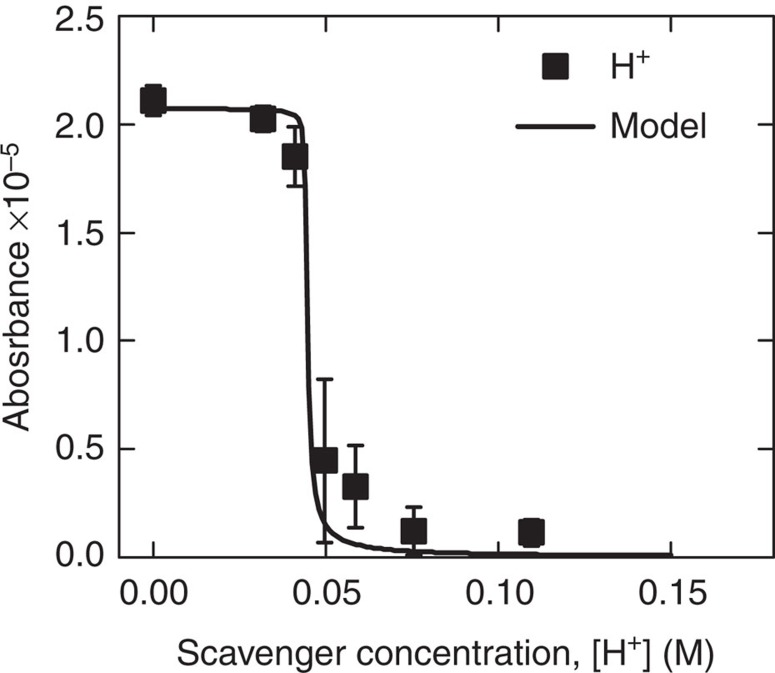
Absorbance behaviour in acidic solutions. Absorbance measurements for (H^+^)_aq_. The solid line is an analytical model that predicts the shift in the decay region to higher concentrations due to a competing reaction with (OH^−^)_aq_. Error bars represent the r.m.s. variance of the absorption signal at 90% confidence.

We also studied the kinetics of the solvated electrons with (H^+^)_aq_ derived from sulfuric acid (H_2_SO_4_) in a background solution of 0.163 M NaClO_4_. The experiments similarly showed a decrease in optical absorption with increasing scavenger concentration (Fig. 3). Note that the magnitude of the signal is significantly greater than the previous measurements. This is because the higher conductivity of the 0.163 M NaClO_4_ solution (15.5 mS cm^−1^) resulted in a larger current density, *j*, which caused the overall magnitude of the optical signal to become larger. Also, note that the critical concentration where the signal begins to decrease has been significantly shifted to higher concentrations, because second-order recombination, reaction (2), produces hydroxide (OH^−^), which will neutralize the acid in the interfacial region via


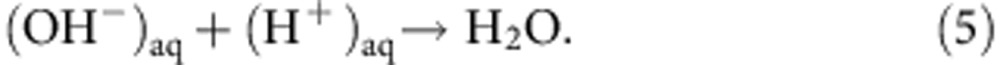


To confirm this effect, we developed a model that included the competing reactions (5) and (4) to predict the local shift (Supplementary Note 7). As shown in Fig. 3, the model qualitatively captures the local depletion of (H^+^)_aq_ scavengers with the use of two fitting parameters, and it can be shown analytically that the shift of the decay region is functionally dependent on the current density, the penetration depth and the diffusion time constant of (H^+^)_aq_ in the interfacial region. A similar, though far less pronounced, shift due to local depletion is also expected for the H_2_O_2_, NO_2_^−^ and NO_3_^−^ scavengers and may explain the non-zero intercepts in Fig. 2c,d.

It is important to note that this approach to generating solvated electrons produces behaviours that do not occur in pulse radiolysis experiments and are entirely unique to this system. In radiolysis, bulk water is ionized to form an (H_2_O^+^)_aq_/(e^−^)_aq_ pair. The (H_2_O^+^)_aq_ quickly reacts to become (H_3_O^+^)_aq_ (equivalent to (H^+^)_aq_). Thus, equal amounts of (H_3_O^+^)_aq_ and (e^−^)_aq_ are produced locally in ‘spurs' that are overall charge neutral^17^. However, in our system, there is a distinct cathodic (negatively charged) region beneath the plasma where electrons solvate and produce an excess of (OH^−^)_aq_ through second-order recombination, similar to electrochemical experiments involving a solid, submerged electrode. This leads to the behaviour observed in Fig. 3 which is distinct from that observed using methods to produce bulk solvated electrons.

## Discussion

In summary, atmospheric-pressure plasmas in contact with aqueous solutions uniquely produce solvated electrons by a completely different mechanism than studied elsewhere. In addition to being inexpensive, compact and free of harmful radiation, this approach provides an inherently rich environment to explore near-surface solvated electron behaviour as well as an unexplored new system for studying a wide range of chemical events at gas–liquid interfaces. However, as shown in this work, there is much to be understood about the nature of near-surface solvated electrons at a plasma–solution interface. In particular, the formation of solvated electrons at a plasma–solution interface opens questions about their behaviour in the presence of strong electric fields, as suggested by the blue-shifted absorption spectrum, and creates the possibility of controlling their concentration and penetration depth by varying the current density.

## Methods

### Experimental apparatus

A modulated d.c. plasma was formed in a background of argon gas at atmospheric pressure by suspending a sharpened stainless steel capillary ∼1 mm above an aqueous electrolyte solution containing a submerged platinum foil counter-electrode (Supplementary Fig. 1). On the basis of our estimated electron penetration depth of the order of nanometres, the optical absorption can be estimated to be only 1 part in 10^5^ using equation (1). Lock-in amplification was, therefore, used to increase the signal to noise by pulsing the plasma between high (*i*_high_=10.9±0.8 mA) and low (*i*_low_=5.6±0.4 mA) currents at a frequency of 20 kHz, and detecting the optical signal at the same frequency. This approach was found to produce a noise baseline on the order of 10^−6^–10^−7^ OD units, depending on the stability of the diode laser. Additional details on the experimental method can be found in the Supplementary Methods.

### Absorption spectrum measurement

A set of seven different continuous wave diode lasers (Thorlabs) with wavelengths of 405, 450, 532, 635, 670, 785 and 850 nm were used along with corresponding band-pass filters to obtain the measured optical absorption spectrum. The optical absorption spectrum was measured in 60 ml solutions of 0.163 M NaClO_4_. At most, three trials were conducted using a given aliquot of solution. A trial consisted of ∼3 min of plasma exposure and optical measurement.

### Scavenger experiments

Four different scavengers were used (NO_2_^−^, NO_3_^−^, H_2_O_2_ and H^+^) and for all cases, a background of NaClO_4_ or NaOH was used to maintain the conductivity of the solution at low scavenger concentrations. It is important to note that neither NaClO_4_ nor NaOH readily react with solvated electrons.

### Chemical preparation

Solutions of 0.163 M NaClO_4_ were prepared by adding 10 g of NaClO_4_ salt (ACS reagent, ≥98.0%, Sigma Aldrich) to 500 ml of deionized (DI) water.

Solutions of NO_2_^−^ were prepared by adding various amounts of NaNO_2_ (99.999% trace metals basis, Fluka, Sigma Aldrich) to 60 ml aliquots of 0.01 M NaOH. Solutions of 0.01 M NaOH were prepared by mixing 50 ml of 0.1 M NaOH (eluent concentrate, Fluka, Sigma Aldrich) with 450 ml of DI water.

Solutions of NO_3_^−^ were prepared by adding various amounts of NaNO_3_ (99.999% trace metals basis, Fluka, Sigma Aldrich) to 60 ml aliquots of 0.01 M NaOH mixed in the manner previously described.

Solutions of H_2_O_2_ were prepared by adding 1.24 g of NaClO_4_ (99.999% trace metals basis, Fluka, Sigma Aldrich) and 10 ml of 30% H_2_O_2_ (Sigma Aldrich) to 490 ml of DI water to obtain final solution concentrations of 0.02 M NaClO_4_ and 0.2 M H_2_O_2_, respectively. These solutions were then diluted with various aliquots of 0.02 M NaClO_4_ to obtain 60 ml aliquots of 0.02 M NaClO_4_ with various H_2_O_2_ concentrations. Solutions of 0.02 M NaClO_4_ were prepared by adding 1.24 g (NaClO_4_)_s_ to 500 ml of DI water. A background NaClO_4_ concentration of 0.02 M was chosen so that the baseline conductivity matched that of a 0.01 M NaOH solution.

Solutions of H^+^ were prepared by adding 10 g of NaClO_4_ (ACS reagent, ≥98.0%, Sigma Aldrich) to 500 ml of 0.1 M H_2_SO_4_ (0.1 M, eluent concentrate, Fluka, Sigma Aldrich). These solutions were then diluted with various aliquots of 0.163 M NaClO_4_ to obtain 60 ml aliquots of 0.163 M NaClO_4_ with various concentrations of H^+^.

## Additional information

**How to cite this article:** Rumbach, P. *et al.* The solvation of electrons by an atmospheric-pressure plasma. *Nat. Commun.* 6:7248 doi: 10.1038/ncomms8248 (2015).

## Supplementary Material

Supplementary Information Supplementary Figures 1-5, Supplementary Table 1, Supplementary Notes 1-7, Supplementary Methods and Supplementary References

## Figures and Tables

**Table 1:** Measured rate constants extrapolated from absorption measurements of solvated electrons at the plasma–solution interface.

**Reaction**	**Measured** ***k*** **(10**^9^ **M**^−1^ **s**^−1^)	**Published** ***k*** **(10**^9^ **M**^−1^ **s**^−1^)	**Published** ***k*****cor. for** ***I***_**S**_ **(10**^9^ **M**^−1^ **s**^−1^)
(e^−^)_aq_+(NO_2_^−^)_aq_→(NO_2_^2−^)_aq_	5.2±1.7	4.1	9.7
(e^−^)_aq_+(NO_3_^−^)_aq_→(NO_3_^2−^)_aq_	7.0±2.6	9.7	17.9
(e^−^)_aq_+(H_2_O_2_)_aq_→(OH)_aq_+(OH^−^)_aq_	14.1±3.1	11.0	11.0

Literature values for bulk reactions obtained from pulse radiolysis experiments as well as values corrected for the ionic strength of our solutions are also shown^17^.

## References

[b1] Hart E. J. & Boag J. W. Absorption spectrum of the hydrated electron in water and in aqueous solutions. J. Am. Chem. Soc. 84, 4090–4095 (1962).

[b2] Hart E. J. & Anbar M. The Hydrated Electron Wiley-Interscience (1970).

[b3] Tabata Y. Pulse Radiolysis CRC Press (1990).

[b4] Wiesenfeld J. M. & Ippen E. P. Dynamics of electron solvation in water. Chem. Phys. Lett. 64, 47–50 (1980).

[b5] Zhu D., Zhang L., Ruther R. E. & Hamers R. J. Photo-illuminated diamond as a solid-state source of solvated electrons in water for nitrogen reduction. Nat. Mater. 12, 836–841 (2013). 23812128 10.1038/nmat3696

[b6] Alexander W. A., Wiens J. P., Minton T. K. & Nathanson G. M. Reactions of solvated electrons initiated by sodium atom ionization at the vacuum-liquid interface. Science 335, 1072–1075 (2012). 22383842 10.1126/science.1215956

[b7] Siefermann K. R. *et al.* Binding energies, lifetimes and implications of bulk and interface solvated electrons in water. Nat. Chem. 2, 274–279 (2010). 21124507 10.1038/nchem.580

[b8] Verlet J. R. R., Bragg A. E., Kammrath A., Cheshnovsky O. & Neumark D. M. Observation of large water-cluster anions with surface-bound excess electrons. Science 307, 93–96 (2005). 15604360 10.1126/science.1106719

[b9] Madarász Á., Rossky P. J. & Turi L. Excess electron relaxation dynamics at water/air interfaces. J. Chem. Phys. 126, 234707 (2007). 17600435 10.1063/1.2741514

[b10] Uhlig F., Herbert J. M., Coons M. P. & Jungwirth P. Optical spectroscopy of the bulk and interfacial hydrated electron from ab initio calculations. J. Phys. Chem. A 118, 7507–7515 (2014). 24576141 10.1021/jp5004243

[b11] Gubkin J. Electrolytische Metallabscheidung an der freien Oberfläche einer Salzlösung. Ann. Phys. 32, 114–115 (1887).

[b12] Goodman J., Hickling A. & Schofield B. The yield of hydrated electrons in glow-discharge electrolysis. J. Electroanal. Chem. Interfacial electrochem. 48, 319–322 (1973).

[b13] Meesungnoen J., Jay-Gerin J.-P., Filali-Mouhim A. & Mankhetkorn S. Low-energy electron penetration range in liquid water. Radiat. Res. 158, 657–660 (2002). 12385644 10.1667/0033-7587(2002)158[0657:leepri]2.0.co;2

[b14] Garrett B. C. *et al.* Role of water in electron-initiated processes and radical chemistry: issues and scientific advances. Chem. Rev. 105, 355–389 (2005). 15720157 10.1021/cr030453x

[b15] Bartels D. M., Takahashi K., Cline J. A., Marin T. W. & Jonah C. D. Pulse radiolysis of supercritical water. 3. Spectrum and thermodynamics of the hydrated electron. J. Phys. Chem. A 109, 1299 (2005). 16833444 10.1021/jp0457141

[b16] Bonin J., Lampre I. & Mostafavi M. Absorption spectrum of the hydrated electron paired with nonreactive metal cations. Radiat. Phys. Chem. 74, 288–296 (2005).

[b17] Park K. Q., Deutsch Z., Li J. J., Oron D. & Weiss S. Single molecule quantum-confined stark effect measurements of semiconductor nanoparticles at room temperature. ACS Nano 7, 10013–10023 (2012). 23075136 10.1021/nn303719m PMC3507316

[b18] Delahay P. Double Layer and Electrode Kinetics Interscience Publishers (1965).

[b19] Buxton G. V., Greenstock C. L., Helman W. P. & Ross A. B. Critical Review of rate constants for reactions of hydrated electrons, hydrogen atoms and hydroxyl radicals (OH/O in aqueous solution. J. Phys. Chem. Ref. Data 17, 513–886 (1988).

[b20] Witzke M., Rumbach P., Go D. B. & Sankaran R. M. Evidence for the electrolysis of water by atmospheric-pressure plasmas formed at the surface of aqueous solutions. J. Phys. D Appl. Phys. 45, 442001 (2012).

[b21] Hare P. M., Price E. A., Stanisky C. M., Janik I. & Bartels D. M. Solvated electron extinction coefficient and oscillator strength in high temperature water. J. Phys. Chem. A 114, 1766–1775 (2010). 20058903 10.1021/jp909789b

[b22] Muroya Y. *et al.* Time-dependent yield of the hydrated electron in subcritical and supercritical water studied by ultrafast pulse radiolysis and Monte-Carlo simulation. Phys. Chem. Chem. Phys. 14, 14325–14333 (2012). 23007023 10.1039/c2cp42260c

[b23] Farouk T., Farouk B., Staack D., Gutsol A. & Fridman A. Simulation of dc atmospheric pressure argon micro glow-discharge. Plasma Sources Sci. Technol. 15, 676–688 (2006).

[b24] Kothnur P., Yuan X. & Raja L. L. Structure of direct-current microdischarge plasmas in helium. Appl. Phys. Lett. 82, 529–531 (2003).

[b25] Turi L. & Rossky P. J. Theoretical studies of spectroscopy and dynamics of hydrated electrons. Chem. Rev. 112, 5641–5674 (2012). 22954423 10.1021/cr300144z

[b26] Larsen R. E., Glover W. J. & Schwartz B. J. Does the hydrated electron occupy a cavity? Science 329, 65–69 (2010). 20595609 10.1126/science.1189588

[b27] Casey J., Kahros A. & Schwartz B. J. To be or not to be in a cavity: the hydrated electron dilemma. J. Phys. Chem. B 117, 14173–14182 (2013). 24160853 10.1021/jp407912k

[b28] Sagar D. M., Bain C. D. & Verlet J. R. R. Hydrated electrons at the water/air interface. J. Am. Chem. Soc. 132, 6917–6919 (2010). 20433171 10.1021/ja101176r

