# Corrigendum: The solvation of electrons by an atmospheric-pressure plasma

**DOI:** 10.1038/ncomms11911

**Published:** 2016-06-06

**Authors:** Paul Rumbach, David M. Bartels, R. Mohan Sankaran, David B. Go

Nature Communications 6 Article number:724810.1038/ncomms8248 (2015); Published: 01192015; Updated 06062016.

We have discovered an error in the original data analysis of our publication, which significantly impacts the model parameters derived from experiment. Specifically, the correction changes our estimations of the average penetration depth *l* and scavenging rate constants, but these new estimates do not change the overall physics conveyed in the Article.

In Supplementary Notes 4 and 5, we reported that the lock-in amplifier measures the AC amplitude of the absorbance signal as the difference between the high and low current states of the plasma (that is, the peak-to-peak amplitude). However, we have now discovered that the lock-in amplifier actually measures the RMS amplitude, which reduces our measurements of the absorbance signal by a factor of 1/(2√2). Furthermore, our signal is essentially a square wave, so the peak amplitude of the first Fourier mode (measured by the lock-in detector) is a factor 4/*π* greater than the peak amplitude of the square wave.

When accounting for both of these, our equation for the measured amplitude, Supplementary Equation 13, was missing a factor of √2/*π*. With this correction, Supplementary Equations 13 and 15 should be


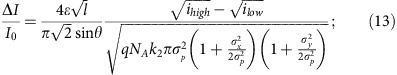



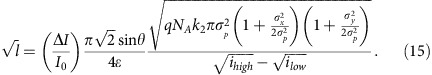


Solving for the average penetration depth *l*, we now estimate a value of *l*=12.3±4.9 nm, which is a factor of *π*^2^/4≈2.47 larger than the originally reported value of 2.5 nm.

Similarly, this affects the calculation of rate constants for scavenger reactions, and Supplementary Equation 21 should be


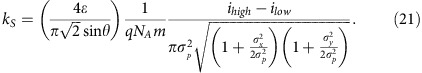


Thus, the ‘Measured *k*' values in Table 1 should be reduced by a factor of √2/*π*. The correct table appears below.

## Figures and Tables

**Table 1:**
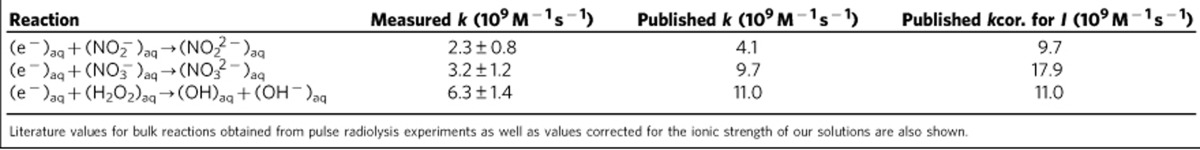
Measured rate constants extrapolated from absorption measurements of solvated electrons at the plasma-solution interface.

