# Supplementary material for: The solvation of electrons by an atmospheric-pressure plasma
Source: Nat Commun. 2015 Jun 19;6:7248. doi: 10.1038/ncomms8248 (PMC4557361; doi:10.1038/ncomms8248)
Supplement: Supplementary Information — Supplementary Figures 1-5, Supplementary Table 1, Supplementary Notes 1-7, Supplementary Methods and Supplementary References [file ncomms8248-s1.pdf]

Supplementary Figures

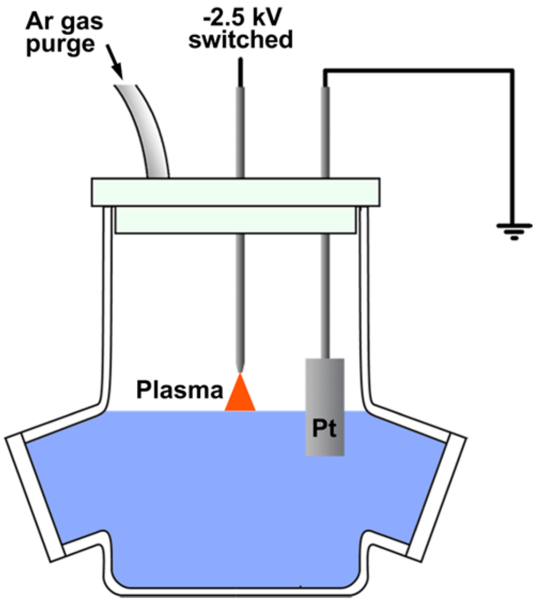

Supplementary Figure 1. Apparatus for measuring solvated electrons using optical absorption. A schematic representation of the plasma electrochemical cell where the plasma is formed between a sharpened capillary and aqueous electrolyte solution with a submerged, grounded platinum (Pt) electrode in an argon (Ar) environment at atmospheric pressure.

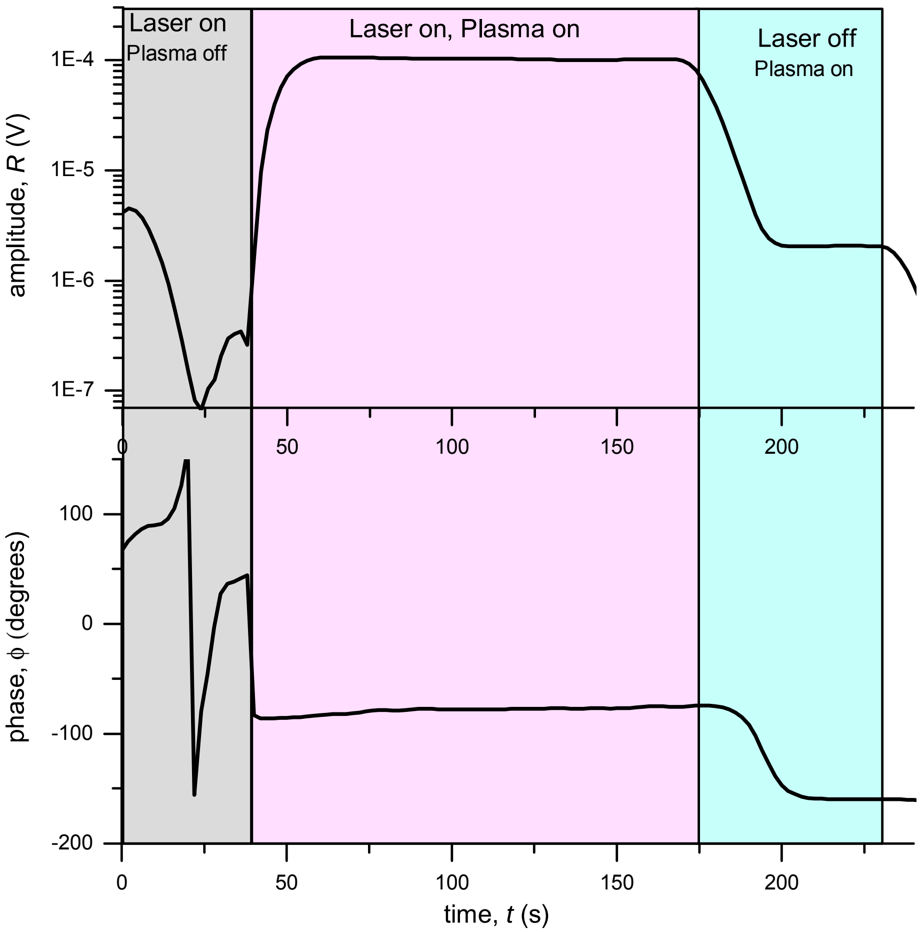

Supplementary Figure 2. Representative raw signal during an absorption measurement. An example of raw data collected from the lock-in amplifier using a 670 nm diode laser and a 163 mM solution of $NaClO_4$. The raw signal for the "Laser on, Plasma on" portion of the data is later averaged and normalized by the total detector voltage for the incident laser, $I_0 \approx 5$ V.

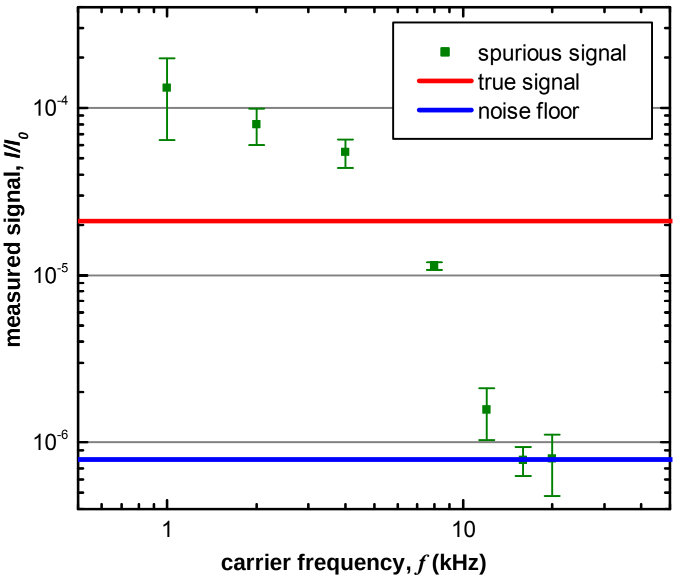

Supplementary Figure 3. Effect of plasma carrier frequency on the measured signal. The experiment is performed using a 670 nm diode laser in a solution containing 150 mM $NaClO_4$ and 490 mM H_2O_2 as an electron scavenger. With such a high scavenger concentration, the signal due to optical absorption by solvated electrons vanishes, and only the spurious signal due to surface vibrations remains. The red solid line indicates our solvated electron absorption signal when operating at 20 kHz (*e.g.*, plasma on, laser on in Supplementary Fig. 2) and the blue solid line indicates our noise level at 20 kHz (*e.g.*, plasma on, laser off in Supplementary Fig. 2). At low frequencies, the spurious signal due to electromechanical vibrations is greater than the true absorption signal, but driving the plasma with an ultrasonic carrier frequency > 10 kHz eliminates this effect.

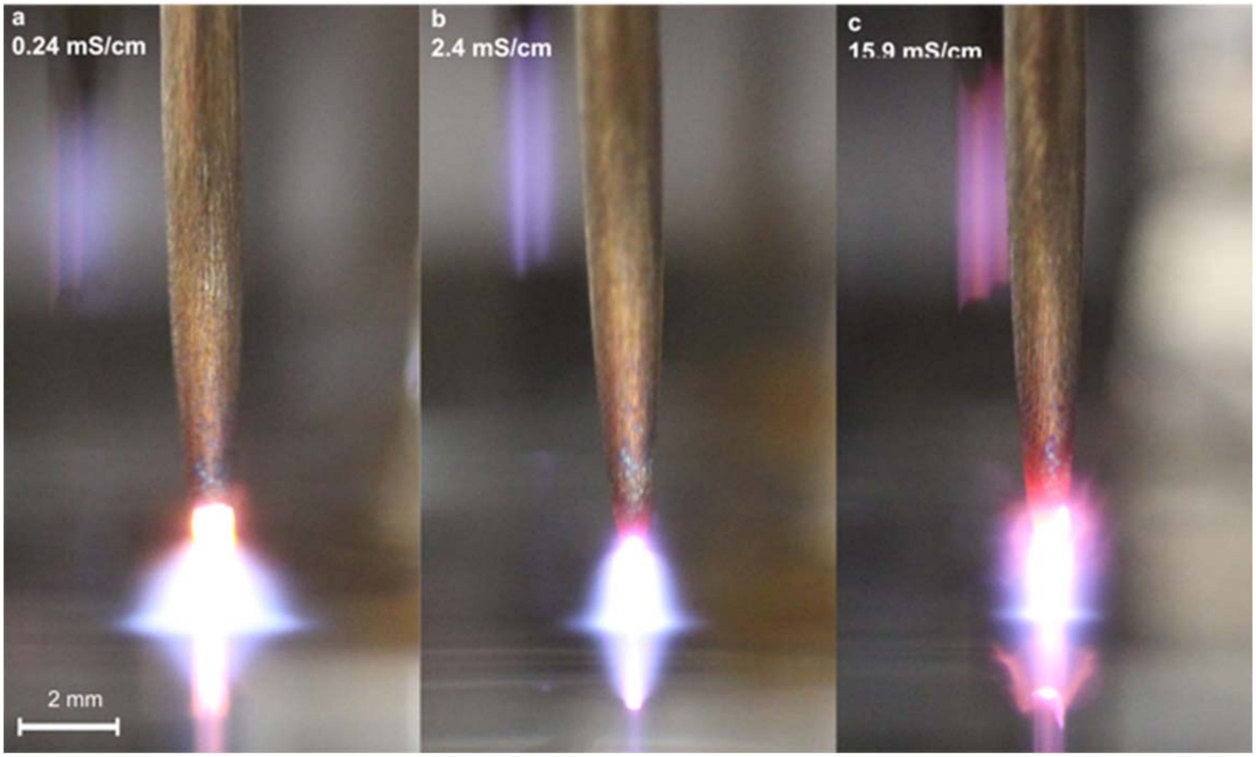

Supplementary Figure 4. Images of plasma at plasma-solution interface. A 10 mA DC argon plasma operated with $NaClO_4$ solutions of varying conductivity (**a**, 0.24 mS cm^{-1}, **b**, 2.4 mS cm^{-1}, **c,** 15.9 mS cm^{-1}), illustrating the variation in the plasma radius, which influences the calculation of the current density.

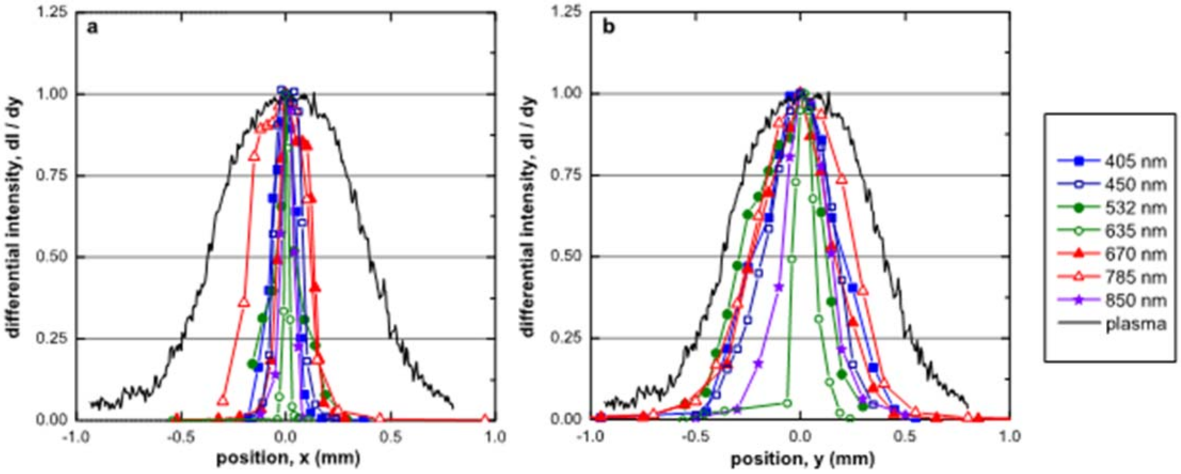

Supplementary Figure 5. Radial laser and plasma intensity profiles. The differential intensity profile of the laser beam measured using a knife-edge method along both axes (**a**, *x*-axis, **b**, *y*-axis) in the horizontal plane compared to the intensity profile of the plasma obtained from digital photographs.

Supplementary Tables

Supplementary Table 1. Measured plasma radii and current densities for various solutions.
**The conductivity of this solution was beyond the range of our probe.*

Solution	Conductivity (mS cm^{-1})	σ_p (mm)	j_{ON} (A m^{-2})	j_{OFF} (A m^{-2})
0.01 M NaOH + 0.330 M $NaNO_2$	N/A*	0.31±0.02	36100±5400	18500±2700
0.01 M NaOH + 0.118 M $NaNO_3$	14.9±0.2	0.36±0.02	26800±3600	13800±1800
0.02 M $NaClO_4$	2.3±0.1	0.46±0.05	16400±3800	8400±1900
0.163 M $NaClO_4$	15.5±0.2	0.40±0.02	21700±2700	11100±1400

Supplementary Note 1 Data Reduction

The detector voltage corresponding to the incident laser intensity, I_0, is used to normalize the amplitude of the recorded signal. For a given experiment, the amplitudes measured in all trials were averaged together and the standard deviation was used to estimate the uncertainty in the optical absorption intensity. For every data point presented in the main manuscript, the experiment was repeated at least six times and the error bars arise from standard uncertainty propagation at 90% confidence.

Supplementary Note 2 Reaction Model

In order to analyze the data and extrapolate physical parameters such as the average penetration depth, we developed a reaction model for the system. Assuming the electrons quickly localize within the first few layers of water, the concentration of solvated electrons as a function of depth $n(x)$ is governed be the reaction-drift-diffusion equation

$$D\frac{d^2n}{dx^2}-v_\mathrm{d}\frac{dn}{dx}=\left(k_\mathrm{S}[\mathrm{S}]+2k_2n\right)n\,, \qquad (1)$$

where D is diffusivity, v_d is a drift velocity induced by the local electric field, [S] is the scavenger concentration, k_S is the rate constant for the scavenging reaction, and k_2 is the rate constant for 2nd order recombination. Note that this equation is nonlinear because of the nature of the 2nd order recombination reaction. Additionally, it is coupled nonlinearly to a similar equation for S(x). Solving the fully coupled, nonlinear system is difficult and beyond the scope of this work. To simplify things, we assume that the local scavenger concentration S(x) is constant and equal to the bulk concentration $[(\mathrm{S})_{aq}]$. We also make a linear approximation for the second order recombination term, yielding

$$D\frac{d^2n}{dx^2} - v_{\mathrm{d}}\frac{dn}{dx} = \left(k_{\mathrm{S}}[\mathrm{S}] + 2k_2 n_0\right)n, \tag{2}$$

where n_0 is the concentration at the average depth of localization, which we set as $x = 0$. Note that this model effectively neglects the first few monolayers of water in which the ballistic electrons become localized. Additionally, the linear approximation *overestimates* the rate of 2nd order recombination. Imposing the boundary condition that $n \to 0$ as $x \to \infty$, we obtain the solution

$$n(x) = n_0 \exp\left(-\frac{x}{l}\right), \tag{3}$$

where l is the average penetration depth. Substituting Supplementary Equation. (3) into Supplementary Equation (2) yields the following equation for l

$$v_{\mathrm{d}} + {D}/{l} = \left(k_{\mathrm{S}}[\mathrm{S}] + 2k_2 n_0\right)l. \tag{4}$$

We then impose a flux boundary condition at $x = 0$

$$\frac{j}{qN_A} = vn(x=0) - D\left.\frac{dn}{dx}\right|_{x=0} = (v_{\mathrm{d}} + {D}/{l})n_0, \tag{5}$$

where Fick's law has been invoked for the diffusive flux. Combining Supplementary Equations. (4) and (5), yields

$$\frac{j}{qN_A l} = \left(k_{\mathrm{S}}[\mathrm{S}] + 2k_2 n_0\right)n_0, \tag{6}$$

which is essentially a volumetric rate balance, where electrons are created at a rate proportional to j and reacted away by scavenging and 2nd order recombination. Note that, if there are no scavengers present, [S] = 0 and we arrive at Eq. (3) of the main manuscript.

To determine the total optical absorbance, we insert Supplementary Equation (3) into Beer's Law and integrate over the depth, x

$$\frac{I}{I_0} = \frac{2\varepsilon}{\sin\theta}\int_0^\infty n_0 \exp\left(-\frac{x}{l}\right)dx = \frac{2\varepsilon l}{\sin\theta}n_0 \tag{7}$$

where I_0 is the incident laser intensity, θ is the angle of incidence relative to the liquid surface, and ε is the extinction coefficient, yielding Eq. (1) of the main manuscript. The end result, given by Supplementary Equations (6) and (7), allows us to treat the interfacial region as a film of thickness, l, with a uniform concentration $[(e^-)_{aq}] = n_0$ along the x-axis. For the remainder of this document, we will utilize this treatment and denote the concentrations n_0 as $[(e^-)_{aq}]$ and [S] as $[(S)_{aq}]$.

It is important to note that Supplementary Equation (2) *overestimates* the rate of 2nd order recombination, so the electrons should penetrate slightly further than suggested by this model. Additionally, this model neglects the first few monolayers of water in which the ~1 eV electrons become localized. Overall, the model *underestimates* the average penetration depth l.

Supplementary Note 3 Plasma/Laser Gaussian Overlap Corrections

Ideally, the laser should be focused to a spot size much less than the plasma radius. However, due to geometric constraints of the system, the laser could only be focused to a spot slightly smaller than the plasma radius as shown in Supplementary Fig. 5. Thus, it becomes necessary to perform a correction for the Gaussian overlap of the two. Additionally, there is considerable uncertainty in the plasma radius and beam spot size and performing this correction is necessary to show how the uncertainty in *overlap* propagates to the extrapolated parameters. We begin by writing Beer's law, Eq. (1), as an overlap integral of the form

$$I = \frac{2\varepsilon l}{\sin\theta} \iint_{\Re^2} I'(x,y)\left[(e^-)_{aq}\right](x,y)\,dx\,dy \cdot \qquad (8)$$

where $I'(x,y)$ is the incident laser intensity profile in units of detector voltage per unit area and $[(e^-)_{aq}](x,y)$ is the concentration of solvated electron at a given position on the plasma-solution interface. As shown in Supplementary Fig. 5, the laser profiles projected on the interface and the plasma emission intensity profile appear to be approximately Gaussian. Thus, we have

$$I'(x,y) = \frac{I_0}{\pi\sigma_x\sigma_y} \exp\left(-\frac{x^2}{\sigma_x^2} - \frac{y^2}{\sigma_y^2}\right), \qquad (9)$$

for the laser intensity profile projected onto the liquid surface, and

$$j(x,y) = \frac{i}{\pi\sigma_p^2} \exp\left(-\frac{x^2}{\sigma_p^2} - \frac{y^2}{\sigma_p^2}\right), \qquad (10)$$

for the current density profile where the Gaussian radii of the beam spot σ_x and σ_y and the plasma radius σ_p can be obtained by curve fitting the data in Supplementary Fig. 5. Note that Supplementary Equations (9) and (10) have been properly normalized such that integrating over the entire *x*-*y* plane yields the total incident intensity I_0 and current *i*, respectively.

Supplementary Note 4 Uncertainty in the Optical Absorption

To estimate the equilibrium concentration of solvated electrons $[(\mathrm{e}^-)_\mathrm{aq}]$ at the interface for a pure $NaClO_4$ solution (*i.e.*, in the absence of scavengers) we assume a simple, steady-state model that balances the rate at which solvated electrons are created by the plasma with the rate at which they are destroyed via second order recombination, Eq. (2). Combining Eq. (3) with Supplementary Equation (10), we obtain the following relationship

$$\left[(\mathrm{e}^-)_\mathrm{aq}\right](x,y)=\sqrt{\frac{i}{\pi\sigma_\mathrm{p}^2 qlN_A k_2}}\exp\left(-\frac{x^2}{2\sigma_\mathrm{p}^2}-\frac{y^2}{2\sigma_\mathrm{p}^2}\right). \quad (11)$$

From this relationship, we can calculate the optical absorbance by substituting Supplementary Equations (9) and (11) into Supplementary Equation (8) and evaluating the integral to yield

$$\frac{I}{I_0}=\frac{2\varepsilon}{\sin\theta}\sqrt{\frac{il}{qN_A k_2\pi\sigma_\mathrm{p}^2\left(1+\sigma_x^2/2\sigma_\mathrm{p}^2\right)\left(1+\sigma_y^2/2\sigma_\mathrm{p}^2\right)}}. \quad (12)$$

The lock-in amplifier measures the modulated amplitude of the signal ΔI, which is essentially the difference in absorption between the *high* and *low* states of the plasma or

$$\frac{\Delta I}{I_0}=\frac{2\varepsilon\sqrt{l}}{\sin\theta}\frac{\sqrt{i_\mathrm{high}}-\sqrt{i_\mathrm{low}}}{\sqrt{qN_A k_2\pi\sigma_\mathrm{p}^2\left(1+\sigma_x^2/2\sigma_\mathrm{p}^2\right)\left(1+\sigma_y^2/2\sigma_\mathrm{p}^2\right)}}. \quad (13)$$

As a series of individual diode lasers were used to map the optical absorption spectrum shown in Fig. 1b, the laser diode and optical filter had to be swapped out for each data point, which introduces a systematic error in the radii of the beam spot σ_x and σ_y, as the different lasers will have different focal properties (i.e. beam divergence, beam waist, and focal length) and the system as a whole will not be aligned exactly as before. To correct for this systematic effect and quantify the uncertainty, we rearrange Supplementary Equation (13) and obtain

$$\varepsilon(\lambda)\propto\frac{\Delta I(\lambda)}{I_0}\sin\theta\frac{\sigma_p\sqrt{\left(1+\sigma_x^2/2\sigma_\mathrm{p}^2\right)\left(1+\sigma_y^2/2\sigma_\mathrm{p}^2\right)}}{\sqrt{i_\mathrm{high}}-\sqrt{i_\mathrm{low}}}, \quad (14)$$

which effectively gives the wavelength dependent extinction coefficient in arbitrary units as a function of measurable quantities. Thus, the data shown in Fig. 1b of the manuscript has been corrected according to the right hand side of Supplementary Equation (14) using the measured parameters for each wavelength and normalized to have an amplitude of unity. The overlaid (red) error bars in Fig. 1b are then calculated as the projection of the uncertainties in each measured parameter $\Delta I/I_0$, σ_x, σ_y, σ_p, i_{high}, i_{low}, and θ onto the right hand side of Supplementary Equation (14).

Supplementary Note 5 Calculation of the Average Penetration Depth

The average penetration depth, l, can be determined by rearranging Supplementary Equation (13) as

$$\sqrt{l} = \left(\frac{\Delta I}{I_0}\right)\frac{\sin\theta}{2\varepsilon}\frac{\sqrt{qN_A k_2 \pi \sigma_p^2 \left(1+\sigma_x^2/2\sigma_p^2\right)\left(1+\sigma_y^2/2\sigma_p^2\right)}}{\sqrt{i_{high}}-\sqrt{i_{low}}}, \qquad (15)$$

and using measured values of $\Delta I/I_0$, σ_x, σ_y, σ_p, i_{high}, i_{low}, and θ. Using the absorption intensity at a wavelength of 670 nm, we obtain a value of l = 2.5 nm. By using the *average* radius of the plasma, as discussed above, and standard uncertainty propagation, we obtain an uncertainty value of ±1.0 nm. However, as previously noted, the radius of the plasma σ_p increases slightly with current in the static limit, and it is unclear to what extent it changes on the 20 kHz timescale of switching. We have estimated this effect and found that it can produce as much as 100% variation in the uncertainty in l, up to ±2.0 nm. Therefore, our estimation of the penetration depth is inherently limited by our ability to accurately determine the current density. This remains an opportunity for future improvements in this measurement technique.

Supplementary Note 6 Extrapolation of Rate Constants

Solvated electrons can reduce a wide variety of cations, anions, and neutral species in aqueous solution. In this work, we will refer to these reactions as *scavenging reactions*, and the species that is reduced by the electron will be referred to as an *electron scavenger*. To estimate the equilibrium concentration of solvated electrons $[(e^-)_{aq}]$ in the presence of a scavenger, we employ Supplementary Equation (16)

$$\frac{j}{lqN_A} = 2k_2\left[(e^-)_{aq}\right]^2 + k_S\left[(S)_{aq}\right]\left[(e^-)_{aq}\right], \qquad (16)$$

where $[(\mathrm{S})_{\mathrm{aq}}]$ is the scavenger concentration and k_{S} is the associated rate constant. Supplementary Equation (S16) can easily be solved using the quadratic formula to obtain

$$\left[(\mathrm{e}^{-})_{\mathrm{aq}}\right]=\frac{k_{\mathrm{S}}\left[(\mathrm{S})_{\mathrm{aq}}\right]}{4k_{2}}\left(\sqrt{1+\frac{8k_{2}j}{lqN_{A}\left(k_{\mathrm{S}}\left[(\mathrm{S})_{\mathrm{aq}}\right]\right)^{2}}}-1\right). \tag{17}$$

For large scavenger concentrations, second order recombination becomes negligible, and in the limit that $\left(k_{\mathrm{S}}\left[(\mathrm{S})_{\mathrm{aq}}\right]\right)^{2} >> {8k_{2}j}/{lqN_{A}}$ along with accounting for Gaussian overlap corrections, we rewrite Supplementary Equation (17) as

$$\left[(\mathrm{e}^{-})_{\mathrm{aq}}\right](x,y)=\frac{i}{\pi\sigma_{\mathrm{p}}^{2}qlN_{A}k_{\mathrm{S}}[(\mathrm{S})_{aq}]}\exp\left(-\frac{x^{2}}{\sigma_{\mathrm{p}}^{2}}-\frac{y^{2}}{\sigma_{\mathrm{p}}^{2}}\right). \tag{18}$$

Combining Supplementary Equation (18) with Beer's law thus produces the normalized optical absorption signal

$$\frac{I}{I_{0}}=\left(\frac{2\varepsilon}{\sin\theta}\right)\frac{1}{qN_{A}k_{S}[(S)_{aq}]}\frac{i}{\pi\sigma_{p}^{2}\sqrt{\left(1+{\sigma_{x}^{2}}/{\sigma_{p}^{2}}\right)\left(1+{\sigma_{y}^{2}}/{\sigma_{p}^{2}}\right)}}. \tag{19}$$

Thus, the normalized optical absorption signal I/I_0 is inversely proportional to scavenger concentration $[(\mathrm{S})_{\mathrm{aq}}]$. As before, the lock-in amplifier measures the difference in absorption between the *high* and *low* states of the plasma

$$\frac{\Delta I}{I_{0}}=\left(\frac{2\varepsilon}{\sin\theta}\right)\frac{1}{qN_{A}k_{\mathrm{s}}[(\mathrm{S})_{\mathrm{aq}}]}\frac{i_{\mathrm{high}}-i_{\mathrm{low}}}{\pi\sigma_{\mathrm{p}}^{2}\sqrt{\left(1+{\sigma_{x}^{2}}/{\sigma_{\mathrm{p}}^{2}}\right)\left(1+{\sigma_{y}^{2}}/{\sigma_{\mathrm{p}}^{2}}\right)}}. \tag{20}$$

Thus, plotting the measured absorption signal $\Delta I/I_0$ as a function of $1/[(\mathrm{S})_{\mathrm{aq}}]$ yields a straight line as shown in Fig. 2 of the main manuscript. Using a linear regression analysis to determine the slope *m* allows us to extrapolate the rate constant

$$k_{\mathrm{S}}=\left(\frac{2\varepsilon}{\sin\theta}\right)\frac{1}{qN_{A}m}\frac{i_{\mathrm{high}}-i_{\mathrm{low}}}{\pi\sigma_{\mathrm{p}}^{2}\sqrt{\left(1+{\sigma_{x}^{2}}/{\sigma_{\mathrm{p}}^{2}}\right)\left(1+{\sigma_{y}^{2}}/{\sigma_{\mathrm{p}}^{2}}\right)}}. \tag{21}$$

Note that this calculation for the rate constant does not account for any variations in the ionic strength of the solution. Our solutions are at fairly high ionic strength I_S and thus in order to compare them to published rate constants from the literature, we apply an ionic strength correction to the published values as

$$\log_{10}\left(\frac{k_{S,corr}}{k_S}\right)=\frac{1.02 Z_S\sqrt{I_S}}{1+\sqrt{I_S}}. \tag{22}$$

Supplementary Note 7 Modeling for Acidic Solutions

Sulfuric acid was used to provide the cationic scavenger H^+. Sulfuric acid has a large equilibrium dissociation constant and essentially completely dissociates into H^+ and HSO_4^- in aqueous solution. The anion HSO_4^- can then further dissociate into H^+ and SO_4^{2-} with a pKa value of 1.92. To calculate the concentration of H^+ in the bulk from the concentration of H_2SO_4, we apply the equilibrium relationship

$$\left[(H^+_{BULK})_{aq}\right]=10^{-1.92}\frac{\left[(HSO_4^-)_{aq}\right]}{\left[(SO_4^{2-})_{aq}\right]}, \tag{23}$$

where the concentration of HSO_4^- and SO_4^{2-} can be determined from

$$\left[(HSO_4^-)_{aq}\right]+\left[(SO_4^{2-})_{aq}\right]=\left[(H_2SO_4)_{aq}\right], \tag{24}$$

which is a statement of the conservation of $(SO_4^{2-})_{aq}$, and

$$\left[(HSO_4^-)_{aq}\right]+\left[(H^+_{BULK})_{aq}\right]=2\left[(H_2SO_4)_{aq}\right], \tag{25}$$

which is a statement of the conservation of $(H^+_{BULK})_{aq}$. Combining Supplementary Equations (23)-(25), yields a quadratic equation for $\left[(H^+_{BULK})_{aq}\right]$, which can be easily solved yielding

$$\left[(H^+_{BULK})_{aq}\right]=\tfrac{1}{2}\left(\left[(H_2SO_4)_{aq}\right]-K_A+\sqrt{\left[(H_2SO_4)_{aq}\right]^2+K_A^2+6K_A\left[(H_2SO_4)_{aq}\right]}\right), \tag{26}$$

where K_A = $10^{-1.92}$ M. Supplementary Equation (26) was used to determine the bulk concentration of H^+ from the concentration of H_2SO_4.

Under acidic conditions, electrons will react with H^+ ions via the scavenging reaction

$$(H^+)_{aq} + (e^-)_{aq} \rightarrow (H\bullet)_{aq}. \tag{27}$$

However, the H• radical produced by reaction Supplementary Equation (27) also serves as an electron scavenger via the reaction

$$(e^{-})_{aq} + (H\bullet)_{aq} \rightarrow H_2 + (OH^{-})_{aq}. \quad (28)$$

Further, OH^{-} produced by second order recombination neutralizes the acid at the interface via reaction (6) in the main manuscript or

$$(OH^{-})_{aq} + (H^{+})_{aq} \rightarrow H_2O. \quad (29)$$

Generally, reaction Supplementary Equation (29) has a rate constant that is nearly an order of magnitude greater than reaction Supplementary Equation (27) such that nearly all of the acid at the interface will be neutralized for low acid concentrations, effectively shifting the acid scavenger data to the right as observed in Fig. 3.

To account for this phenomenon, it is necessary to have rate balance equations for $(e^{-})_{aq}$, H^{+}, OH^{-}, and H•. For electrons, the rate balance is

$$\frac{j}{lqN_A} = 2k_2\left[(e^{-})_{aq}\right]^2 + k_{20}\left[(H^{+})_{aq}\right]\left[(e^{-})_{aq}\right] + k_{21}\left[(H\bullet)_{aq}\right]\left[(e^{-})_{aq}\right], \quad (30)$$

where the creation of electrons by the plasma has been balanced with second order reaction rates for reactions (2), Supplementary Equation (27), and Supplementary Equation (28). For protons H^{+}, the rate balance is

$$\frac{1}{\tau_H}\left(\left[(H^{+})_{aq}\right] - \left[(H^{+}_{BULK})_{aq}\right]\right) = k_{22}\left[(H^{+})_{aq}\right]\left[(OH^{-})_{aq}\right] + k_{20}\left[(H^{+})_{aq}\right]\left[(e^{-})_{aq}\right], \quad (31)$$

where local depletion of H^{+} by reactions Supplementary Equation (27) and Supplementary Equation (29) is balanced by diffusion of H^{+} from the bulk into the interfacial reaction zone. Note that the zero dimensional form used for diffusion is analogous to Newton's law of cooling, where τ_H is the time constant associated with the diffusion of H^{+}. For the hydroxide ion OH^{-}, we have

$$2k_2\left[(e^{-})_{aq}\right]^2 + k_{21}\left[(H\bullet)_{aq}\right]\left[(e^{-})_{aq}\right] = k_{22}\left[(H^{+})_{aq}\right]\left[(OH^{-})_{aq}\right] + \frac{1}{\tau_{OH}}\left[(OH^{-})_{aq}\right], \quad (32)$$

where the creation of OH^{-} by reactions (2) and Supplementary Equation (28) is balanced by the acid-base neutralization reaction Supplementary Equation (29) and the diffusion of OH^{-} out of the interfacial region. As before, a form for diffusion analogous to Newton's law of cooling has

been used, where τ_{OH} is the time constant associated with diffusion of OH⁻. Lastly, assuming all the H• radicals produced by Supplementary Equation (27) quickly react via Supplementary Equation (28) before diffusing out of the interfacial region, we have the simple rate balance

$$k_{20}\left[(\mathrm{H^+})_{aq}\right]\left[(\mathrm{e^-})_{aq}\right]=k_{21}\left[(\mathrm{H\bullet})_{aq}\right]\left[(\mathrm{e^-})_{aq}\right]. \tag{33}$$

Overall, we seek to find the local concentration of solvated electrons as a function of the bulk acid concentration $\left[(H^+_{\mathrm{BULK}})_{aq}\right]$. We begin by substituting Supplementary Equation (33) into Supplementary Equations (30) and (32) to obtain

$$\frac{j}{lqN_A}=2k_2\left[(\mathrm{e^-})_{aq}\right]^2+2k_{20}\left[(\mathrm{H^+})_{aq}\right]\left[(\mathrm{e^-})_{aq}\right], \tag{34}$$

and

$$2k_2\left[(\mathrm{e^-})_{aq}\right]^2+k_{20}\left[(\mathrm{H^+})_{aq}\right]\left[(\mathrm{e^-})_{aq}\right]=k_{22}\left[(\mathrm{H^+})_{aq}\right]\left[(\mathrm{OH^-})_{aq}\right]+\frac{1}{\tau_{OH}}\left[(\mathrm{OH^-})_{aq}\right]. \tag{35}$$

Supplementary Equation (34) can be solved using the quadratic formula to obtain

$$\left[(\mathrm{e^-})_{aq}\right]=\frac{k_{20}\left[(\mathrm{H^+})_{aq}\right]}{2k_2}\left(\sqrt{1+\frac{8k_2 j}{lqN_A\left(2k_{20}\left[(\mathrm{H^+})_{aq}\right]\right)^2}}-1\right). \tag{36}$$

yielding the *local* electron concentration as a function of the *local* H^+ concentration. However, in the experiments, we measure $[(e^-)_{aq}]$ as a function of the *bulk* concentration of H^+. Thus, it is necessary to obtain the local concentration $[(H^+)_{aq}]$ in terms of the bulk concentration $\left[(\mathrm{H^+_{BULK}})_{aq}\right]$. Combining Supplementary Equations (31), (34), and (35), the system reduces to a 4th order polynomial for $[(H^+)_{aq}]$, which has been omitted for brevity. Solving this polynomial numerically using a root finder, we obtain a result that matches the data rather well using $\tau_H \sim \tau_{OH} \sim 10^{-6}$ s as a fitting parameter, as shown in Fig. 3 of the main manuscript.

Overall, we observe the same scaling as before, where the optical absorption scales inversely with scavenger concentration for high concentrations. However, the decay region of the curve has been shifted significantly to the right. We can analytically explain this by making one simple approximation. For large acid concentrations, we assume all the hydroxide is neutralized before it diffuses out of the interfacial region. Thus, Supplementary Equation (35) becomes

$$2k_2\left[(\mathrm{e^-})_{aq}\right]^2=k_{22}\left[(\mathrm{H^+})_{aq}\right]\left[(\mathrm{OH^-})_{aq}\right]-k_{20}\left[(\mathrm{H^+})_{aq}\right]\left[(\mathrm{e^-})_{aq}\right]. \tag{37}$$

Combining this expression with Supplementary Equations (31) and (34) yields

$$\frac{1}{\tau_{\mathrm{H}}}\left(\left[(\mathrm{H}^{+})_{\mathrm{aq}}\right]-\left[(\mathrm{H}_{\mathrm{BULK}}^{+})_{\mathrm{aq}}\right]\right)=\frac{j}{qlN_{A}}. \tag{38}$$

Rearranging Supplementary Equation (38) produces an effective local H^+ concentration as affected by acid neutralization at the interface or

$$\left[(\mathrm{H}^{+})_{\mathrm{aq}}\right]=\left[(\mathrm{H}_{\mathrm{BULK}}^{+})_{\mathrm{aq}}\right]-\frac{j\tau_{\mathrm{H}}}{qlN_{A}}. \tag{39}$$

Thus, the curve given by Supplementary Equation (36) and the data in Fig. 3 are shifted to the right by an amount $j\tau_H/qlN_A$ for high concentrations of acid. The raw data appears to be shifted by an amount of about 0.03 M, and using measured values for l, we found that values of $\tau_{\mathrm{H}} \sim 10^{-6}$ s and $j \sim 10^{3}$ A m^{-2} fit the data well. Note that this model is overly simplistic and requires τ_{H} as a fitting parameter as well as a current density, j, to match the magnitude of the spectrum. More detailed modeling is required to match the data without any fitting parameters.

Supplementary Methods

Plasma Source

Supplementary Fig. 1 shows a schematic of the experimental set-up for generating solvated electrons with an atmospheric-pressure plasma at the surface of an aqueous solution, which adopts a configuration similar to a conventional electrochemical cell[1]. A direct current (DC), atmospheric-pressure microplasma is formed with a sharpened stainless steel capillary tube (180 μm inner diameter, 5 cm length, Restek Corp.) as the cathode, suspended 1–2 mm above the liquid surface. A submerged piece of platinum (Pt) foil serves as the counter-electrode, *i.e.,* anode. The headspace of the vessel is continuously purged with argon (Ar) at a flow rate of 300 sccm, controlled and measured using a rotameter (1.4 SLM air/20 SCCM water, Omega Engineering Inc.), so that the reactor vessel is filled with pure Ar. This prevents the formation of reactive plasma species that typically form in atmospheric air, such as NO_x and O_3, which readily dissolve into solution and alter its chemical composition[1].

The plasma is generated by applying a negative DC bias (-2.5 kV) to the sharpened capillary using a high voltage power supply (PS325, Stanford Research Systems) with the Pt anode connected to an earth ground. As discussed in the ensuing sections, for the purposes of lock-in amplification, the plasma current is modulated between a high (i_{high}) and low (i_{low}) state at a known carrier frequency. In order to monitor the plasma current, the voltage drop across a 8.1 kΩ resistor connected in series with the cathode was measured with a high voltage differential probe (THPD0100, Tektronix) and an oscilloscope (DPO 2024B, Tektronix). The

measured values were i_{high} = 10.9±0.8 mA and i_{low} = 5.6±0.4 mA.

Optical Setup

Solvated electrons were detected using optical absorption spectroscopy in a total internal reflection geometry. A diode laser is mounted in line with an iris and 50 mm lens in an optical cage system. The entire optical assembly is fixed to a goniometer, so it may be rotated to a desired angle. The laser beam enters through one of the angled windows shown in Supplementary Fig. 1, reflects off the solution surface beneath the gas interface, exits through the opposing angled window, and hits a photodetector. For all experiments, the optical assembly was fixed at an angle of 17° ± 1° relative to the surface of the solution. Because the angle is less than the critical angle for total internal reflection, the laser beam does not propagate into the headspace of the reactor and is entirely reflected toward the detector, excluding the small amount of light absorbed by solvated electrons at the interface. The total internal reflection conditions ensure that the entire measured signal is due only to absorbance by solvated electrons, as opposed to other spurious (e.g. random reflection) losses. This approach produces a very low background noise floor with the plasma off so that we can detect absorbance with a high signal-to-noise ratio. The detector and reactor vessel are attached to kinematic mounts for alignment. The entire setup is mounted to an optical table to minimize the effects of vibration.

Lock-in Amplification

Because the solvated electrons form in such a thin layer at the interface (~nm), the optical path is short, and the absorbance is weak. Thus, it is necessary to use lock-in amplification to obtain a signal-to-noise ratio sufficiently high to resolve their absorbance. To achieve this, we used an amplitude modulation strategy whereby the plasma current was modulated between high (i_{high}) and low (i_{low}) values using a solid-state relay circuit. Modulating the current also modulates the concentration of solvated electrons at the interface, which in turn modulates the amount of optical absorption. In short, an insulated gate bipolar junction transistor (IGBT, IXGH10N300, IXYS Corp.) is used to rapidly switch the flow of current through an additional ballast resistor. The rate of switching—or carrier frequency—is set by a grounded function generator (B8085, Protek), which is coupled to the IGBT using an optical isolator (TLP250, Toshiba). Output from the photodetector is fed into a lock-in amplifier (SR830, Stanford Research Systems), which filters out all noises sources that are not at the carrier frequency. The TTL output from the function generator driving the plasma switching circuit was used as the reference signal for the lock-in amplifier to determine the detection frequency. The lock-in amplifier was set to have a time constant of 3 s, and the carrier frequency was 20 kHz, which is discussed in detail in the following section.

A time series of the amplitude and phase of the optical absorption signal from the lock-in amplifier were recorded remotely on a computer using LabView software. Data was collected for 1 min with the laser on and the plasma off to ensure the noise floor of the laser was sufficient. The plasma was then turned on yielding a signal due to optical absorption. After approximately

3 min, the laser was turned off to ensure the signal was not due to any electromagnetic noise from the plasma. A representative example of the raw data from the lock-in amplifier is shown in Supplementary Fig. 2.

The true signal due to optical absorption by solvated electrons can be seen in the portion of Supplementary Fig. 2 labeled "Laser on, Plasma on". This signal consistently has a phase between -75° and -100°. In the "Laser on, Plasma off" portion of Supplementary Fig. 2, the incident laser intensity I_0 is shown to have an inherent noise signal between 10^{-7} to 10^{-6}. This noise does not have a definite phase and can be averaged out. For samples containing a sufficiently large concentration of electron scavengers, the optical signal in the "Plasma on, Laser on" portion of Fig. 2 should theoretically vanish. In practice, the signal drops by an order of magnitude to 1 part in 10^6, and the phase becomes incoherent, implying that the true noise floor of the measurement is near 10^{-6}.

Spurious Signals and Noise Sources

The lock-in amplifier is ineffective at filtering noise sources at the 20 kHz carrier frequency. For example, modulating the current at 20 kHz also modulates the optical emission of the plasma, which can be picked up by the optical detector. This spurious signal is *not* filtered by the lock-in. A simple remedy for this is to mount an optical band-pass filter corresponding to the laser diode over the detector. Additionally, the modulated plasma behaves like an antenna, emitting electromagnetic (EM) radiation at 20 kHz, which gets picked up by the detector circuit. By Mounting the detector in a thick aluminum box and using double-walled coaxial cables, we suppressed the spurious signal due to EM pick-up to less than 10^{-6}. The signal due to EM interference can be seen in the "Laser off, Plasma on" portion of Supplementary Fig. 2. Because it has a definite phase, the EM signal can be subtracted off from the data if necessary.

Another spurious signal arises from the electromechanical vibration of the liquid surface due to electromagnetic Maxwell stresses induced by the plasma. Modulating the plasma current between the high and low states effectively modulates the amount of electrical charge on the liquid surface, causing it to vibrate up and down and act as a lens, scattering the incident laser at the carrier frequency. It was ultimately discovered that modulating the plasma at a frequency faster than the inertial response of surface capillary waves sufficiently eliminated this problem. To demonstrate this, the experiment was performed with a 670 nm laser in a solution containing 150 mM $NaClO_4$ and 490 mM H_2O_2 as an electron scavenger. With such a high scavenger concentration, the concentration of solvated electrons drops below any optically detectable limit, leaving behind only the spurious signal and noise. As shown in Supplementary Fig. 3, the spurious signal due to electromechanical vibrations of the liquid surface quickly falls off near 10 kHz. By operating at 20 kHz, we ensured that spurious signals due to electromechanical vibration did not ultimately impact our findings.

Current Density Measurement

Obtaining an accurate value of the current density, j, at the interface is critical for making extrapolations from measured data in this experiment. Assuming the current density is simply the current, i, divided by the area of the plasma-solution interface $\pi\sigma_p^2$, it becomes necessary to measure both the current, i, and the effective plasma radius, σ_p. We note that these measurements are inherently difficult, and as such the calculation of the current density is the largest source of uncertainty in our analysis of the penetration depth and the reaction rate constants.

To measure the effective plasma radius, σ_p, a series of at least 10 images were taken of the plasma while switching at 20 kHz using a digital camera (Canon EOS Rebel T3i) at a shutter speed of 1/160 s. Radial intensity profiles were extracted from these images, and the effective radius of the plasma was then determined by applying a Gaussian curve fit to the intensity profile.

The conductivity of the solution greatly affects the plasma radius and the overall stability of the plasma. If the conductivity of the solution is too low, the interface becomes charged and begins to vibrate at the carrier frequency, resulting in a spurious signal. Additionally, for a fixed current, i, increasing the conductivity of the solution decreases the radius of the plasma. This causes the current density, j, to increase such that, higher solution conductivity results in a higher flux of electrons at the interface, causing the overall optical absorption to increase. Supplementary Fig. 4 shows representative images of the plasma for different conductivity solutions, and Supplementary Fig. 5 shows a typical intensity profile. Measured values of σ_p and subsequent calculated values for j_{high}, and j_{low} are listed in Supplementary Table 1 for the various solutions used in these experiments. Solution conductivity was measured using a conductivity probe (ECTestr 11 dual-range, pin-style pocket conductivity tester, Oakton), and as noted above, the measured currents were i_{high} = 10.9±0.8 mA and i_{low} = 5.6±0.4 mA for every case studied.

We note that it is common for the plasma radius to increase with current in the static limit[2]. However, it is unclear to what extent it changes on the 20 kHz timescale of switching as we lack the high speed imaging equipment to temporally resolve the fast switching rate. Essentially, the images taken of the plasma while switching provide an *average* radius of the plasma. Using this average radius instead of the transient radii of the plasma in its *high* and *low* states is the main source of error in the extrapolated values of the average penetration depth, l, and the effective rate constants. Also, it is unclear how well the emission intensity profile of the plasma corresponds to its current density profile.

Laser Spot Size

In order to analyze the optical absorption data, it is ultimately necessary to know the overlap between the plasma and the laser. To assess this overlap, we measured the laser spot size and profile. The laser is focused onto the interface with a 50 mm lens (LA1131, Thorlabs for visible wavelengths and AC254-050-B, Thorlabs for near infrared). The intensity profile of the spot incident on the interface was measured using a knife-edge method[3]. In short, the reaction chamber was replaced with a razor blade mounted coplanar with the usual location of the liquid

surface. The kinematic mounts were used to "eclipse" the laser spot with the edge of the razor. Measuring the detector voltage as a function of razor position yields the integral of the beam intensity profile. Thus, numerically differentiating this data yields a good estimate of the light intensity profile. Intensity profiles along both axes are shown in Supplementary Fig. 5 as well as a representative measurement of the optical emission profile of the plasma.

Supplementary References

1. Rumbach, P., Witzke, M., Sankaran, R. M., & Go, D. B., Decoupling interfacial reactions between plasmas and liquids: Charge transfer vs plasma neutral reactions. *J. Am. Chem. Soc.* **135**, 16264-16267 (2013).
2. Raizer, Y. P. *Gas Discharge Physics* (Springer-Verlag, Berlin, 1987), pp. 174.
3. Arnaud, J. A. *et al.* Technique for fast measurement of Gaussian laser beam parameters. *Appl. Opt.* **10**, 2775-2776 (1971).
